# Long-Term Health Outcomes of Huntington Disease and the Impact of Future Disease-Modifying Treatments: A Decision-Modeling Analysis

**DOI:** 10.1212/CPJ.0000000000200340

**Published:** 2024-08-15

**Authors:** Gregory F. Guzauskas, Sarah J. Tabrizi, Jeffrey D. Long, Astri Arnesen, Jamie L. Hamilton, Daniel O. Claassen, Lorraine R. Munetsi, Shahid Malik, Idaira Rodríguez-Santana, Talaha M. Ali, Frank Zhang

**Affiliations:** The Comparative Health Outcomes (GFG), Policy, and Economics Institute, Department of Pharmacy, University of Washington, Seattle, WA and HCD Economics, Daresbury; Huntington's Disease Centre (GFG, LRM, SM, IRS), UCL Queen Square Institute of Neurology, London, United Kingdom; The University of Iowa Health Care (SJT), Iowa City, IA; European Huntington Association (JDL), Moerbeke Waas, Belgium; Clinical Department (AA), CHDI Management/CHDI Foundation, Princeton, NJ; Department of Neurology (JLH), Vanderbilt University Medical Center, Nashville, TN; HCD Economics (DOC), Daresbury, United Kingdom; and uniQure Inc. (TMA, FZ), Lexington, MA.

## Abstract

**Background and Objectives:**

Disease-modifying treatments (DMTs) such as gene therapy are currently under investigation as a potential treatment for Huntington disease (HD). Our objective was to estimate the long-term natural history of HD progression and explore the potential efficacy impacts and value of a hypothetical DMT using a decision-analytic modeling framework.

**Methods:**

We developed a health state transition model that separately analyzed 40-year-old individuals with prefunctional decline (PFD, HD Integrated Staging System [HD-ISS] stage <3, total functional score [TFC] 13), active functional decline Shoulson and Fahn category 1 (SF1, HD-ISS stage 3, TFC 13-11), and SF2 (HD-ISS stage 3, TFC 10-7). Three-year outcomes from the TRACK-HD longitudinal study were linearly extrapolated to estimate the long-term health outcomes and costs of each population. For PFD individuals, we used the HD-ISS to predict the onset of functional decline. HD costs and quality-adjusted life years (QALYs) were estimated over a lifetime horizon by applying health state–specific costs and utilities derived from a related HD burden-of-illness study. We then estimated the long-term health impacts of hypothetical DMTs that slowed or delayed onset of functional decline. We conducted sensitivity analyses to assess model uncertainties.

**Results:**

The expected life years for 40-year-old PFD, SF1, and SF2 populations were 20.46 (95% credible range [CR]: 19.05–22.30), 13.93 (10.82–19.08), and 10.99 (8.28–22.07), respectively. The expected QALYs for PFD, SF1, and SF2 populations were 15.93 (14.91–17.44), 8.29 (6.36–11.79), and 5.79 (4.14–12.91), respectively. The lifetime costs of HD were $508,200 ($310,300 to $803,700) for the PFD population, $1.15 million ($684,500 to $1.89 million) for SF1 individuals, and $1.07 million ($571,700 to $2.26 million) for SF2 individuals. Although hypothetical DMTs led to cost savings in the PFD population by delaying the cost burdens of functional decline, they increased costs in SF1 and SF2 populations by prolonging time spent in expensive progressive HD states.

**Discussion:**

Our novel HD-modeling framework estimates HD progression over a lifetime and the associated costs and QALYs. Our approach can be used for future cost-effectiveness models as positive DMT clinical trial evidence becomes available.

## Introduction

Huntington disease (HD) is a rare, inherited, neurodegenerative disorder characterized by progressive motor symptoms including involuntary choreatic movements as well as cognitive, behavioral, and psychiatric changes.^1,2^ The disease is caused by an increase in the number of CAG repeats in the DNA sequence of exon 1 of the *HTT* gene.^3^ The overall prevalence of HD is increasing,^4^ and the economic and humanistic burden associated with the condition is substantial.^5,6^ Recent research estimates that annual costs associated with HD in the United States ranges from $6,600 to $30,300 per year depending on the stage of progression.^5^ Furthermore, there is limited evidence available providing a comprehensive assessment of the clinical, economic, and humanistic burden of HD by disease stage and on a large scale.

Currently, there is no cure for HD, and available treatments do not alter the course of disease progression. Medications such as tetrabenazine (Xenazine) and deutetrabenazine (Austedo) may lessen movement disorder (chorea) symptoms^7^ while antidepressants and antipsychotic drugs can be used in tandem but may also have an effect on chorea.^8^ Treatments typically evolve over the course of the disease, depending on individual individuals' overall treatment goals.

Potential disease-modifying treatments (DMTs) such as gene therapy (GTx), antisense oligonucleotides (ASOs), and small molecule–based therapeutic approaches aim to provide long-term health benefits for people with HD by slowing down, arresting, or even reverting disease progression.^9^ Once clinical trial evidence becomes available, health economic research will be necessary to quantify the long-term costs and health outcomes for health care decision makers. In anticipation of future DMTs for HD, we aimed to estimate the long-term natural history of HD progression and explore the efficacy and potential value of hypothetical DMTs to treat HD using a decision-analytic modeling framework.

## Methods

### Overall Approach

We developed our decision-analytic modeling framework after consultation with an advisory board of independent HD researchers, including HD clinicians, HD study investigators, HD patient advocates, and a health economist, among others. We used annual model cycles, a lifetime horizon, and a US payer perspective (i.e., focused on direct medical care costs [in 2023 US dollars^10^] only) as well as a modified societal perspective incorporating indirect medical costs and productivity loss. We discounted all model outcomes by 3% per year to reflect their present value^11^; undiscounted values were also estimated. The model was developed in Microsoft Excel. Our report adheres to the Consolidated Health Economic Evaluation Reporting Standards best practices checklist for economic analysis.^12^

**Figure 1:**
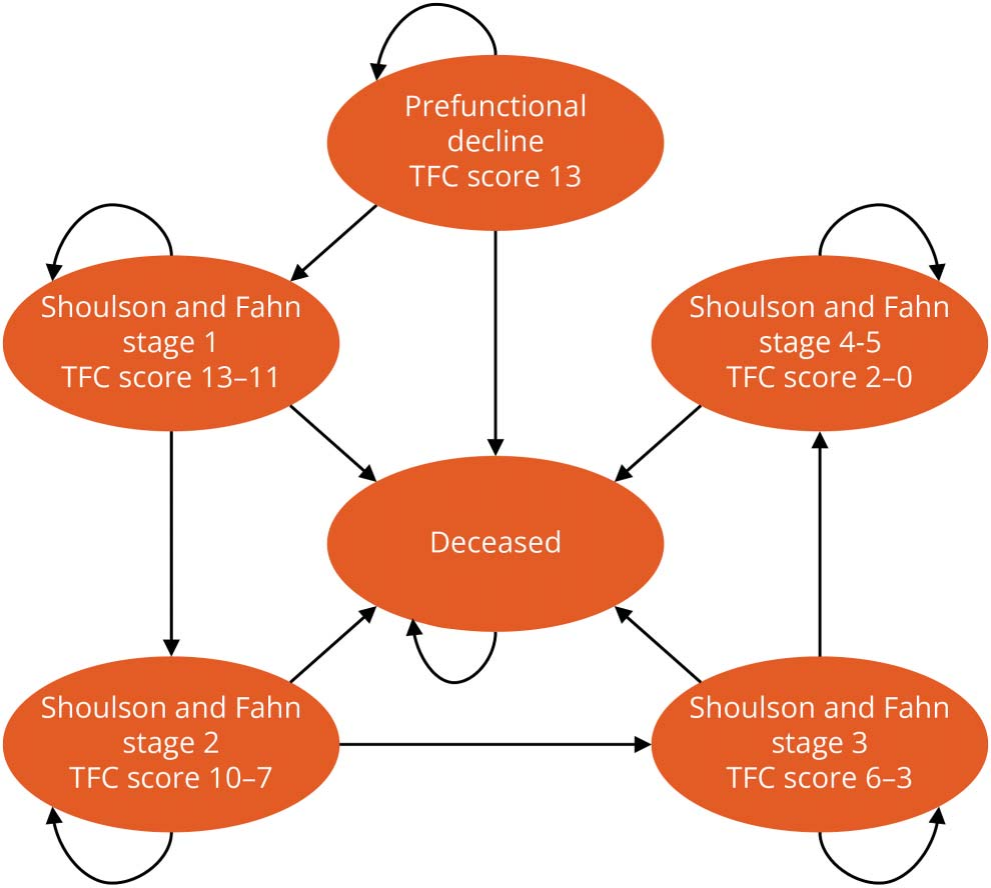
Model Schematic People with HD can enter the model in either the PFD, SF1, or SF2 health states, depending on the modeled population. As indicated by the straight arrows, they may then transition to more progressed health states at a constant rate of progression based on a simple linear regression of TRACK-HD^16,17^ data. Alternatively, the curves arrows indicate they may remain in their previous health state. They may also transition to death at any time by US background mortality.^15^ In hypothetical comparisons with future disease-modifying treatments, the constant rate of transitions to more progressed health states is both arrested for a given amount of time and progression is slowed by a given rate ratio. PFD = prefunctional decline; SF2 = Shoulson and Fahn stage 2 (TFC 10-7); SF1 = Shoulson and Fahn stage 1 (TFC 13-11).

The primary driver of modeled HD progression was the total functional capacity (TFC) score, a standardized scale used to assess capacity to work, handle finances, perform domestic chores and self-care tasks, and live independently.^13^ The TFC scale ranges from 13 (normal) to 0 (severe disability). The Shoulson and Fahn (SF) system broadly categorizes the TFC scale into 5 stages (SF1: 13-11, SF2: 10-7, SF3: 6-3, SF4: 2-1, and SF5: 0).^14^ We used a multistate Markov model approach to annually transition individuals through each distinct TFC score (depending on their starting score when entering the model), but as shown in Figure 1, we applied health state payoffs (costs and utilities) to grouped TFC scores according to the Shoulson and Fahn stages for which these payoffs were previously estimated. We assumed that a TFC score of zero (i.e., SF5) was equivalent to death, and modeled individuals could also die at any time from secular death derived from US life tables.^15^ All modeled outcomes were summed over the lifetime horizon to estimate each population's total cost, life years, and quality-adjusted life years (QALYs).

### Standard Protocol Approvals, Registrations, and Patient Consents

This decision-analytic modeling study is based solely on the summary outcomes of aggregated data from previous studies and does not involve individual patient data. Therefore, ethical approvals, trial registrations, or patient consents were not required.

### HD Progression Modeling

We modeled 3 hypothetical 40-year-old populations: one in prefunctional decline (PFD; i.e., before symptom onset) and 2 (SF1 and SF2) in active functional decline. These populations entered the model in the PFD, SF1, or SF2 health states (Figure 1). Long-term TFC score progression was modeled using baseline, 12-month, and 36-month TFC scores for the SF1 and SF2 populations (Table 1) from the TRACK-HD study,^16,17^ a multinational prospective observational study that examined the clinical and biological findings of disease progression in individuals with PFD HD and early-stage HD. We chose TRACK-HD as the basis of long-term projections because it had publicly available, rigorously compiled results that were straightforward to incorporate into the model.

**Table 1:** Model Parameters

Parameter	Base case	Lower	Upper	Probabilistic distribution	Source
General model settings					
Patient age (all populations)	40				Assumption
Annual discount rate	3%				Neumann^11^
Sensitivity analyses, range % +/-	25%				Assumption
Number of probabilistic analysis simulations	5,000				Assumption
Prefunctional decline population assumptions					
CAG repeats	40				Advisory board
Total functional capacity (TFC) score	13				Advisory board
Total motor score (TMS)^a^	2				Advisory board
Symbol digit modalities test (SDMT) score^a^	50				Advisory board
Natural history outcomes for SF1 (TFC 13-11)					
Total functional capacity (TFC) score baseline	12.2				TRACK-HD^16^
TFC 12-mo Δ	−0.910	−1.360	−0.620	Normal	TRACK-HD^16^
TFC 36-mo Δ	−1.989	Correlated		TRACK-HD^17^	
Annual TFC transition probability^b^	0.651	Varies with SLR^c^		SLR-derived^c^	
Natural history outcomes for SF2 (TFC 10-7)					
Total functional capacity (TFC) score baseline	8.7				TRACK-HD^16^
TFC 12-mo Δ	−0.440	−0.780	−0.130	Normal	TRACK-HD^16^
TFC 36-mo Δ	−1.974	Correlated		TRACK-HD^17^	
Annual TFC transition probability	0.669	Varies with SLR^c^		SLR-derived^c^	
Health state utilities					
Premanifest	0.86	0.84	0.87	Beta	Jiang^27^
Shoulson and Fahn stage 1 (TFC 11–13)	0.78	0.72	0.85	Beta	Rodríguez-Santana^28^
Shoulson and Fahn stage 2 (TFC 7–10)	0.66	0.59	0.73	Beta	Rodríguez-Santana^28^
Shoulson and Fahn stage 3 (TFC 3–6)	0.54	0.48	0.61	Beta	Rodríguez-Santana^28^
Shoulson and Fahn stage 4–5 (TFC 0–2)	0.22	0.11	0.32	Beta	Rodríguez-Santana^28^
Direct medical costs					
Shoulson and Fahn stage 1 (TFC 13-11)	$13,183	$5,723	$20,644	Log-normal	Rodríguez-Santana^29^
Shoulson and Fahn stage 2 (TFC 10-7)	$16,549	$3,786	$29,311	Log-normal	Rodríguez-Santana^29^
Shoulson and Fahn stage 3 (TFC 6-3)	$20,189	$5,404	$34,974	Log-normal	Rodríguez-Santana^29^
Shoulson and Fahn stage 4 (TFC 2-1)	$48,031	$18,983	$77,080	Log-normal	Rodríguez-Santana^29^
Shoulson and Fahn stage 5 (TFC 0)	$25,217	$0	$55,668	Log-normal	Rodríguez-Santana^29^
Societal costs					
Shoulson and Fahn stage 1 (TFC 13-11)	$30,691	$11,333	$50,048	Log-normal	Rodríguez-Santana^29^
Shoulson and Fahn stage 2 (TFC 10-7)	$41,676	$15,390	$67,962	Log-normal	Rodríguez-Santana^29^
Shoulson and Fahn stage 3 (TFC 6-3)	$110,142	$38,727	$185,849	Log-normal	Rodríguez-Santana^29^
Shoulson and Fahn stage 4 (TFC 2-1)	$58,561	$1,724	$115,398	Log-normal	Rodríguez-Santana^29^
Shoulson and Fahn stage 5 (TFC 0)	$36,409	$1,072	$71,746	Log-normal	Rodríguez-Santana^29^

a For low-education (high school only) population.

b Also used for the prefunctional decline (PFD) population once progression commences.

c SLR = simple linear regression of TRACK-HD baseline, 1-year, and 3-year TFC scores.

We fit a simple linear regression model to results of the 2 TRACK-HD populations to estimate constant rates of TFC progression, which we then used to derive the annualized transition probabilities of HD-affected populations through the 14 possible TFC health states. The PFD population was assumed to progress at the same constant rate as the SF1 population at the onset of active functional decline. We checked our assumption of a constant rate of TFC progression by analyzing the Enroll-HD database (PDS5, obtained in March 2023), a clinical research platform and the world's largest observational HD study, including data on more than 20,000 participants in Europe, North America, Australasia, and Latin America.^18,19^ Data used in this work were generously provided by the participants in the Enroll-HD study and made available by CHDI Foundation, Inc. Enroll-HD is a global clinical research platform designed to facilitate clinical research in Huntington disease. Core data sets are collected annually from all research participants as part of this multicenter longitudinal observational study. Data are monitored for quality and accuracy using a risk-based monitoring approach. All sites are required to obtain and maintain local ethical approval.

**Figure 2:**
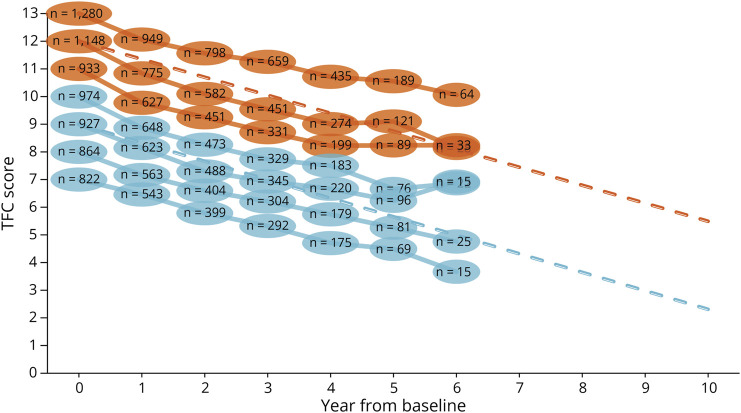
Natural History of HD The ovals are the observed means from Enroll-HD^19^ at each visit, with the sample size indicated at each time point. Dashed lines indicate the simple linear regression extrapolations based on 3-year TRACK-HD^16,17^ average TFC progression for motor-manifest HD (UHDRS DCL = 4). n = number of individuals per average score in Enroll-HD; TFC = total functional capacity.

Among Enroll-HD participants with a Unified Huntington's Disease Rating Scale (UHDRS) diagnostic confidence level (DCL) of 4 (i.e., the rater has ≥99% confidence motor abnormalities that are unequivocal signs of the disease),^20^ at baseline, we observed a linear trend in TFC scores over 7 years of follow-up in both SF1 and SF2 subpopulations (Figure 2). We then verified this trend with the advisory board of independent HD researchers.

### Symptom Onset in Prefunctional Decline Population

**Figure 3:**
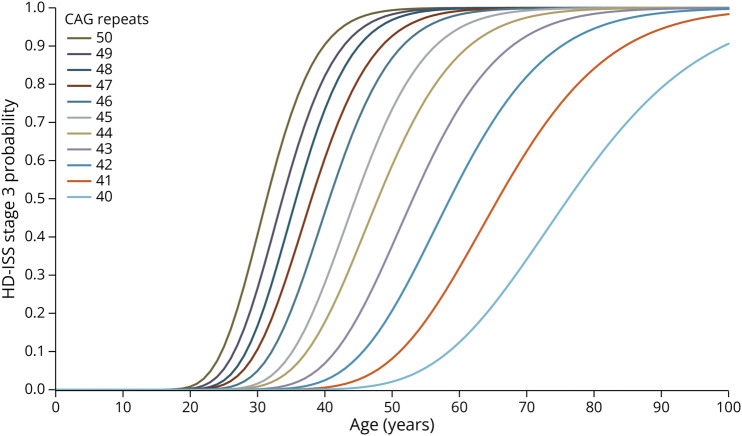
Parametric Curve Fits to HD-ISS Stage 3 Cumulative Probabilities, by Age and Number of CAG Repeats For each 100% PFD population of a given age entering the model, we calculated a reweighted probability of functional decline onset per modeled year tied to the number of remaining years until age 100. PFD = prefunctional decline.

We used the recently introduced HD Integrated Staging System (HD-ISS) to estimate the timing of functional decline onset among the PFD population (eFigure 1). The HD-ISS is an evidence-based research framework for HD that includes criteria to define a HD case and a staging system that encompasses the full progression of the disease from birth to death, including indicators of underlying pathophysiology (stage 1), a detectable clinical phenotype (stage 2), and eventual decline in function (stage 3).^21^ To model the functional decline onset, we fit parametric curves to the HD-ISS–reported cumulative probabilities of stage 3 transition, which were stratified by age and number of CAG repeats,^21^ using an ordinary least squares approach (Figure 3). With each 100% PFD population of a given age entering the model, we then calculated a reweighted probability of functional decline onset per modeled year tied to the number of remaining years until age 100. We then estimated a pooled TFC progression curve from 60 subcohorts (i.e., 100-year-old maximum modeled age minus the modeled population age of 40) experiencing onset as predicted by the HD-ISS over 60 years, to capture variation in the timing of onset (eFigures 2–9). At the onset of active functional decline, each subcohort followed the same extrapolation-based HD progression as SF1 individuals, as described above.

We also modeled the prognostic index normalized for HD (PIN_HD_)^22^ as an alternative method for estimating the onset of functional decline. The PIN_HD_ was originally developed as a progression index anchored to the onset of DCL = 4 and was developed by HD investigators from the PREDICT-HD,^23^ COHORT,^24^ TRACK-HD,^16,17^ and REGISTRY observational studies.^25^ However, PIN_HD_ also seems to be highly relevant for tracking HD signs of the PFD population.^22^ PIN_HD_ is computed as the weighted composite of the UHDRS total motor score (TMS), symbol digit modalities test (SDMT), and the (adjusted) product of CAG repeats and age at baseline and then is normalized for interpretability.

With this PIN_HD_ approach, we performed a scenario analysis on a representative, low-education PFD population with CAG repeats = 40, TMS = 2, SDMT = 50, and an average age of 40 based on published estimates^26^ and in consultation with the advisory board of independent HD researchers. The PIN_HD_ increased with age with each annual model cycle; thus, the transition probability to active functional decline HD increased over time. Similar to our approach for HD-ISS, we then estimated a pooled TFC progression curve from 60 subcohorts experiencing onset as predicted by the PIN_HD_ over 60 years, and each subcohort similarly followed the extrapolation-based HD progression as SF1 individuals at the onset of active functional decline, as described above.

### Quality-of-Life Parameters

The PFD population, for which we modeled a spectrum of symptom onset timing over 60 years based on the HD-ISS, was assigned a health state utility equivalent to the general US population that was obtained from a US-based EQ-5D-5L study of population norms.^27^ For the active functional decline health states (SF1-SF4), we used health state utilities obtained from the EQ-5D-5L outcomes of a related retrospective, cross-sectional study of the HD burden of illness conducted between September 2020 and May 2021 in France, Germany, Italy, Spain, the United Kingdom, and the United States,^28^ which found that EQ-5D-5L utilities in individuals with HD worsened with disease progression. In this study, the anxiety and depression dimension was the main driver of poor EQ-5D-5L scores in early and moderate stages while mobility, self-care, and usual activity dimensions were the main drivers of lower scores in later stages.

### Cost Parameters

We similarly derived health state–specific costs from the same burden-of-illness study,^29,30^ which estimated annual direct medical costs, direct nonmedical costs, and indirect costs associated with HD. Direct medical costs included those related to hospitalizations, medications, consultation visits, tests and examinations, over-the-counter (OTC) and self-medication, physical aids and equipment, residential care, and professional caregiving services. Direct nonmedical costs included travel costs, transfer payments (state support), and alternative therapies. Indirect costs assessed the impact of HD on patient and caregiver work productivity based on hours worked per week, absenteeism, informal care costs, and early retirement. For actively employed people with HD and caregivers, productivity loss was quantified as number of days missed from work because of HD in the past 3 months multiplied by the country average salary per day. For those unable to work because of HD, an opportunity cost was assigned based on one year of average salary per country. We did not model treatment for other, co-occurring conditions.

### Model Analysis

We calculated total life years, QALYs, and direct medical plus indirect costs for HD natural history over modeled individuals' remaining lifetime. Base case results were calculated using the base case estimate for each model parameter. A probabilistic sensitivity analysis (PSA) was conducted in which all model parameters were simultaneously and randomly varied according to assigned probability distributions (TFC scores: normal distributions; utilities: beta distributions; costs: log-normal distributions; Table 1) over 5,000 simulations. Based on the PSA, Bayesian 95% credible ranges (CRs) were calculated for each model result. Of note, the simple linear regression for progression was dynamically recalculated in sensitivity analyses, wherein the 12-month and 36-month TRACK-HD parameters were correlated and the 12-month parameters could take on values within their study-reported 95% CIs.

We conducted an exploratory analysis to evaluate the cost and health implications of hypothetical DMTs for HD, employing efficacy assumptions pertaining to the extent further HD progression is delayed and decelerating the rate of disease progression post-onset. We demonstrate these potential impacts in each of the 3 modeled populations with 3 efficacy scenarios (eFigures 10–12): (1) DMT A delays further functional decline by 3 years and reduces the rate of TFC progression by 25% (rate ratio applied to the constant TFC progression rate = 0.75); (2) DMT B delays further functional decline by 2 years and reduces the rate of TFC progression by 15% (rate ratio = 0.85); and (3) DMT C delays further functional decline by 1 year and reduces the rate of TFC progression by 5% (rate ratio = 0.95). As with the primary analysis of HD natural history, we performed probabilistic analyses to assess the impacts and potential drivers of uncertainty in model parameters on the exploratory results. Finally, we performed one-way sensitivity analyses to assess the impacts of uncertainty in model parameters on the incremental results of each hypothetical DMT vs HD natural history. In one-way sensitivity analysis, one parameter at a time is varied to its plausible low and high values while keeping all other parameters constant.

### Data Availability

We have made the Microsoft Excel model file available as an online supplement to the article. This file contains all input parameters (all of which are from aggregate, not individual patient, data) and outcome calculations. The references for all data sources are indicated throughout the model, and all sources are available online.

## Results

### HD Natural History Outcomes

**Figure 4:**
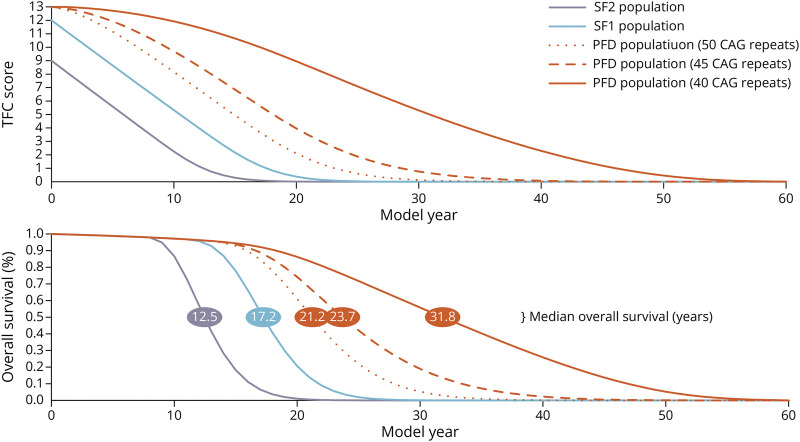
HD Natural History: Average TFC Score and Overall Survival for Modeled 40-Year-Old HD Populations TFC = total functional capacity; PFD = prefunctional decline; SF2 = Shoulson and Fahn stage 2 (TFC 10-7); SF1 = Shoulson and Fahn stage 1 (TFC 13-11).

The lifetime extrapolation for the 40-year-old, 40-CAG repeat PFD population resulted in 20.46 discounted remaining life years (95% CR: 19.05–22.30) and 32.52 undiscounted remaining life years (95% CR: 29.27–37.64) (Table 2). The predicted median survival time for the PFD, SF1, and SF2 populations was 31.8, 17.2, and 12.5 remaining years, respectively (Figure 4). Applying utility weights to the life years resulted in 15.93 discounted QALYs (95% CR: 14.91–17.44) and 24.53 undiscounted QALYs (95% CR: 22.41–28.18). The discounted, HD-related costs of standard care ($126,700 [95% CR: $74,000–$203,400]) and societal burden ($381,500 [95% CR: $203,500–$659,300]) resulted in a combined lifetime cost of $508,200 (95% CR: $310,300–$803,700); the undiscounted, combined lifetime cost was $1.04 million (95% CR: $605,700–$1.70 million). We also found that higher CAG repeat numbers, which conferred greater likelihood of age-adjusted functional decline onset, increased lifetime costs while life years and QALYs tended to decrease because higher CAG repeat populations spent less time in the PFD health state and transitioned more quickly into the expensive, lower quality-of-life progression health states (eTables 1–4).

**Table 2:** Model Results for Natural History of HD

	^a^Prefunctional decline population		Shoulson and Fahn stage 1 population		Shoulson and Fahn stage 2 population	
	Base case	95% credible range	Base case	95% credible range	Base case	95% credible range
Discounted (3% annually) outcomes						
Total costs	$508,185	$310,322 to $803,738	$1,148,001	$684,463 to $1,890,836	$1,074,852	$571,656 to $2,261,835
Standard care	$126,658	$74,000 to $203,425	$286,932	$166,641 to $498,574	$258,505	$129,220 to $551,468
Shoulson and Fahn stage 1	$18,960	$8,712 to $38,018	$38,836	$18,472 to $82,347	—	—
Shoulson and Fahn stage 2	$39,340	$11,851 to $98,648	$84,221	$25,233 to $222,523	$69,631	$20,493 to $264,494
Shoulson and Fahn stage 3	$36,168	$12,221 to $82,408	$83,656	$28,898 to $190,609	$95,754	$28,613 to $251,721
Shoulson and Fahn stage 4–5	$32,189	$13,403 to $58,582	$80,219	$34,694 to $151,660	$93,120	$31,316 to $188,030
Societal cost	$381,527	$203,487 to $659,313	$861,069	$442,721 to $1,541,503	$816,347	$382,772 to $1,845,534
Shoulson and Fahn stage 1	$44,140	$16,606 to $97,709	$90,411	$35,792 to $204,997	—	—
Shoulson and Fahn stage 2	$99,074	$41,222 to $206,498	$212,104	$89,412 to $446,572	$175,360	$67,452 to $611,320
Shoulson and Fahn stage 3	$197,317	$83,990 to $389,646	$456,388	$180,050 to $930,370	$522,390	$191,413 to $1,232,080
Shoulson and Fahn stage 4–5	$40,996	$2,747 to $199,446	$102,165	$7,005 to $491,406	$118,596	$6,641 to $583,754
Total QALYs	15.93	14.91 to 17.44	8.29	6.36 to 11.79	5.79	4.14 to 12.91
Prefunctional decline	12.11	11.81 to 12.60	—	—	—	—
Shoulson and Fahn stage 1	1.13	0.83 to 1.72	2.31	1.67 to 3.76	—	—
Shoulson and Fahn stage 2	1.57	1.21 to 2.09	3.35	2.53 to 4.91	2.77	1.90 to 8.33
Shoulson and Fahn stage 3	0.97	0.80 to 1.11	2.25	1.76 to 2.84	2.57	1.81 to 4.30
Shoulson and Fahn stage 4–5	0.15	0.08 to 0.23	0.38	0.20 to 0.58	0.44	0.20 to 0.72
Total life years	20.46	19.05 to 22.30	13.93	10.82 to 19.08	10.99	8.28 to 22.07
Undiscounted outcomes						
Total costs	$1,036,543	$605,715 to $1,696,369	$1,547,533	$857,986 to $2,982,127	$1,319,022	$654,363 to $3,577,753
Standard care	$258,686	$149,724 to $429,579	$389,033	$213,816 to $735,896	$322,687	$155,796 to $858,465
Shoulson and Fahn stage 1	$31,779	$14,418 to $67,388	$40,322	$18,223 to $86,478	—	—
Shoulson and Fahn stage 2	$73,329	$22,107 to $185,441	$99,912	$30,590 to $276,835	$73,726	$20,550 to $335,615
Shoulson and Fahn stage 3	$77,382	$25,203 to $171,617	$118,639	$40,640 to $295,642	$118,028	$34,886 to $366,745
Shoulson and Fahn stage 4–5	$76,196	$32,714 to $142,244	$130,160	$54,658 to $278,364	$130,933	$44,442 to $327,548
Societal cost	$777,857	$401,087 to $1,402,095	$1,158,500	$554,107 to $2,415,677	$996,334	$427,038 to $2,883,906
Shoulson and Fahn stage 1	$73,982	$28,437 to $168,350	$93,869	$36,011 to $221,717	—	—
Shoulson and Fahn stage 2	$184,673	$77,807 to $406,172	$251,619	$104,449 to $592,225	$185,672	$71,806 to $769,436
Shoulson and Fahn stage 3	$422,160	$172,554 to $863,018	$647,243	$256,740 to $1,465,711	$643,908	$225,247 to $1,885,819
Shoulson and Fahn stage 4–5	$97,042	$6,688 to $459,831	$165,769	$11,591 to $875,066	$166,754	$9,200 to $940,510
Total QALYs	24.53	22.41 to 28.18	10.18	7.29 to 16.78	6.73	4.56 to 20.51
Prefunctional decline	17.28	16.82 to 18.05	—	—	—	—
Shoulson and Fahn stage 1	1.89	1.36 to 3.07	2.40	1.69 to 4.10	—	—
Shoulson and Fahn stage 2	2.92	2.14 to 4.33	3.98	2.82 to 6.74	2.94	1.98 to 10.79
Shoulson and Fahn stage 3	2.08	1.59 to 2.60	3.19	2.28 to 5.07	3.17	2.08 to 8.04
Shoulson and Fahn stage 4–5	0.36	0.19 to 0.55	0.61	0.32 to 1.07	0.62	0.28 to 1.32
Total life years	32.52	29.27 to 37.64	17.82	12.89 to 28.43	13.17	9.35 to 37.36

Abbreviations: QALY = quality-adjusted life year; TFC = total functional score.

a Prefunctional decline population: age = 40, CAG repeats = 40; results for prefunctional decline individuals with 45 and 50 CAG repeats are available in eAppendix 1.

Compared with the PFD population, the SF1 population resulted in fewer expected life years (13.93 [95% CR: 10.82–19.08], discounted) and QALYs (8.29 [95% CR: 6.36–11.79], discounted) and greater total cost ($1.15 million [95% CR: ($684,500–$1.89 million], discounted) because of HD onset and progression being underway. The SF2 population had the lowest expected life years (10.99 [95% CR: 8.28–22.07], discounted) and QALYs (5.79 [95% CR: 4.14–12.19], discounted) but had marginally lower costs than the SF1 population ($1.07 million [95% CR: ($571,700–$2.26 million]), primarily because of lower life expectancy and thus less time spent in expensive health states, given they began the model in a more advanced state of disease progression.

### Exploratory Disease-Modifying Treatment Outcomes

DMTs resulted in gains in life years and QALYs for all 3 modeled populations compared with natural history, driven by the assumed delay of onset/further progression and the assumed rate ratios applied to the rate of progression once onset began (Table 3). The life year and QALY gains were lower for the PFD population (1.48 and 1.42, respectively) compared with the SF1 (4.04 and 3.13, respectively) and SF2 (4.17 and 2.67, respectively) populations. This was due to (1) the staggered symptom onset (driven by the HD-ISS) over 60 years in the PFD population compared with the homogeneous, already progressed SF1 and SF2 populations and (2) the immediate, universal application of the assumed delay of onset/further progression in the already progressing SF1 and SF2 populations. However, while there were potential standard care and societal cost savings resulting from DMTs observed in the PFD population, standard care and societal costs were generally increased compared with natural history in the SF1 and SF2 populations because of prolonged time spent in the more expensive, lower quality-of-life health states. This finding was evident in the majority of 95% CRs from the PSA, which tended to span cost-saving and cost-additive results. In one-way sensitivity analyses, the model was most sensitive to the progression risk ratio and the delay of progression onset, the rate of progression derived from TRACK-HD outcomes, and societal costs (eFigures 13–21).

**Table 3:** Exploratory Analysis Incremental Results: Hypothetical Disease-Modifying Treatments vs HD Natural History

	DMT A vs HD natural history		DMT B vs HD natural history		DMT C vs HD natural history	
	Base case	95% credible range	Base case	95% credible range	Base case	95% credible range
Prefunctional decline population^a^ (discounted)						
Incremental costs	−$35,220	−$142,904 to $20,972	−$21,808	−$92,426 to $25,927	−$14,335	−$69,357 to $33,443
Standard care	−$9,086	−$34,024 to $4,748	−$5,646	−$22,640 to $6,805	−$3,651	−$17,455 to $8,079
Societal cost	−$26,134	−$117,076 to $18,106	−$16,161	−$74,125 to $20,410	−$10,684	−$54,983 to $26,386
Incremental QALYs	1.42	0.74 to 2.03	0.92	0.29 to 1.57	0.40	−0.24 to 1.09
Prefunctional decline	1.41	1.24 to 1.65	0.94	0.79 to 1.15	0.46	0.32 to 0.66
Shoulson and Fahn stage 1	0.13	−0.11 to 0.41	0.06	−0.16 to 0.34	0.00	−0.21 to 0.27
Shoulson and Fahn stage 2	0.04	−0.24 to 0.24	0.01	−0.22 to 0.23	−0.02	−0.24 to 0.22
Shoulson and Fahn stage 3	−0.12	−0.39 to −0.01	−0.07	−0.25 to 0.01	−0.04	−0.14 to 0.03
Shoulson and Fahn stage 4–5	−0.04	−0.09 to −0.01	−0.02	−0.06 to −0.01	−0.01	−0.04 to 0.00
Incremental life years	1.48	0.47 to 2.29	0.97	0.10 to 1.79	0.40	−0.49 to 1.30
Shoulson and Fahn stage 1 population (discounted)						
Incremental costs	$188,245	−$34,489 to $395,879	$115,352	−$64,427 to $308,235	$40,184	−$135,263 to $215,048
Standard care	$52,599	−$3,679 to $108,738	$32,723	−$11,960 to $83,852	$12,107	−$33,590 to $58,901
Societal cost	$135,646	−$38,269 to $313,565	$82,630	−$59,226 to $239,628	$28,077	−$106,944 to $166,203
Incremental QALYs	3.13	1.61 to 4.61	1.96	0.53 to 3.54	0.82	−0.62 to 2.43
Shoulson and Fahn stage 1	2.70	2.11 to 3.55	1.75	1.23 to 2.54	0.82	0.32 to 1.48
Shoulson and Fahn stage 2	0.40	−0.27 to 1.07	0.20	−0.41 to 0.91	0.02	−0.59 to 0.72
Shoulson and Fahn stage 3	0.05	−0.41 to 0.30	0.02	−0.28 to 0.29	−0.02	−0.32 to 0.27
Shoulson and Fahn stage 4–5	−0.02	−0.16 to 0.01	−0.01	−0.09 to 0.02	−0.01	−0.06 to 0.02
Incremental life years	4.04	1.65 to 6.18	2.52	0.32 to 4.87	1.01	−1.23 to 3.42
Shoulson and Fahn stage 2 population (discounted)						
Incremental costs	$279,724	−$72,371 to $550,523	$168,799	−$72,088 to $403,636	$62,875	−$132,939 to $274,364
Standard care	$72,437	−$21,048 to $171,036	$44,476	−$16,205 to $118,729	$17,327	−$29,767 to $74,145
Societal cost	$207,287	−$67,763 to $432,404	$124,323	−$67,643 to $317,933	$45,548	−$109,101 to $214,394
Incremental QALYs	2.67	1.00 to 3.92	1.67	0.32 to 2.95	0.71	−0.44 to 1.99
Shoulson and Fahn stage 2	2.40	1.62 to 3.42	1.53	0.80 to 2.49	0.70	0.00 to 1.62
Shoulson and Fahn stage 3	0.26	−0.92 to 0.68	0.13	−0.46 to 0.58	0.01	−0.47 to 0.46
Shoulson and Fahn stage 4–5	0.01	−0.21 to 0.07	0.01	−0.12 to 0.07	0.00	−0.09 to 0.06
Incremental life years	4.17	0.74 to 6.16	2.59	0.12 to 4.71	1.07	−1.02 to 3.22

Abbreviations: QALY = quality-adjusted life year; TFC = total functional score.

a Prefunctional decline population: age = 40, CAG repeats = 40; results for prefunctional decline individuals with 45 and 50 CAG repeats are available in eAppendix 1.

## Discussion

We developed a decision model to track the natural history of HD progression and associated costs, survival, and quality of life of 3 40-year-old HD populations over their remaining lifetime. Our results support previous HD burden-of-illness study findings that people with HD incur substantial cost and quality-of-life burdens that increase as HD progresses. It is important to note that our model can be used to explore these relationships and to target stages of disease where the health impacts of future DMTs are most likely to be cost effective. In time, the model can be adapted to incorporate clinical trial findings for future DMTs.

Our natural history results for the 3 modeled HD populations expectedly showed that people with HD who are at earlier stages of disease have a longer life expectancy and greater quality of life but lower lifetime cost compared with later stages of disease, although all modeled individuals who do not die from other causes (background mortality) would eventually progress through each expensive stage of HD before death. This finding was due to the annualized 3% discount applied to direct and indirect costs and health outcomes over time, a standard health economics method that applies greater weight to values nearesr to the present than to the future.^11^ In essence, each population's quality of life was greatest at the outset of the model when it was time-weighted the most and declined as time progressed but was time-weighted progressively less. The opposite is true for cost in that it is time-weighted the most at its lowest values and time-weighted the least at its highest values. To address potential confusion, we presented both discounted and undiscounted natural history results, with the latter showing greater cost and QALY parity among the modeled patient populations, given later-stage outcomes were not time-weighted and thus equivalent.

Our exploratory analysis of hypothetical HD treatments that slow and/or arrest HD progression demonstrates that cost savings and QALY gains may be procured from increased survival time in less progressed health states, but that prolonging survival within expensive, low quality-of-life stages of late disease may lead to overall increases in cost. A previous HD-modeling analysis by Albin and Burke reached similar conclusions compared with our study.^31^ They used a Markov chain Monte Carlo approach to simulate the effects of potential therapies with effects on time to onset of HD and survival with HD and found that a delay in symptom onset (0.7–8.1 years depending on patient age and CGG repeat length) was associated with a 5.2-5.3-year increase in life years spent with HD. Our model indicated that the hypothetical treatment effect with the greatest potential to derail potential cost savings was the rate ratio parameter applied to the constant rate of progression once onset began, which did result in overall QALY gains for all 3 patient populations but became more costly in populations initiating treatment when at lower TFC scores. The combination of these results seems to indicate that treating people with HD with active functional decline likely achieves good value up to a point followed by diminishing returns, a finding that may affect treatment discontinuation decisions and the eventual shift from DMT to supportive and palliative care.

Of note, the hypothetical DMT efficacies used in our exploratory analysis are highly speculative. Furthermore, the DMTs currently under development vary in mechanism of action (small molecule, ASO, gene therapy), route of administration (oral, intrathecal, intraparenchymal), and once vs recurrent therapy; these contingencies make it difficult at this time to assign a DMT price in our exploratory analysis. Future cost-effectiveness models based on settled regimens and clinical trial efficacy data should estimate the value-based price of each DMT under accepted willingness to pay per QALY threshold for a given country.

Our analysis had several limitations. First, our projections for TFC progression are uncertain, given follow-up data from TRACK-HD are limited to 3 years^17^ and our assumption that declines in TFC score progress at a constant rate thereafter. We addressed this limitation by varying the 12-month and 36-month TFC outcomes in sensitivity analyses, which provides a range of potential progression trajectories, and by applying variable rate ratios to the rate of progression in our exploratory analyses. We also validated our assumption of a constant progression rate through comparison with the Enroll-HD database.^18,19^ Nonetheless, future research with longer-term follow-up of people may identify distinct progression trajectories that do not follow a linear path.

Second, our analysis focused on the TFC score because it is commonly used to assess an individual's ability to function in daily life, the availability of longitudinal data, and the availability of cost and utility values estimated within the broader TFC-based Shoulson and Fahn categories. However, the TFC score is limited in that it does not capture the emotional, relational, or psychological stressors associated with HD, including in individuals who have yet to experience motor symptom onset, and has been shown to be insensitive to quality of life, particularly neuropsychiatric symptoms.^32,33^ Based on the new HD Integrated Staging System, brain deterioration precedes cognitive and motor manifestation, which precedes functional decline.^21^ The first functional decline tends to be a modification at one's occupation because of a disease effect (motor and/or cognitive). Neuropsychiatric symptoms can have a profound impact on both the person with HD and caregivers, especially when characterized by aggression.^34^ These same burdens are particularly consequential to the families and children of individuals with HD.^32,35^ Such problems can also affect activities of daily living that are indexed by the TFC; however, the TFC does not consider specifically how neuropsychiatric symptoms might affect daily functioning. Thus, the role of neuropsychiatric symptoms in progression is complicated because there is not a clear tracking with progression in other domains, such as motor. Furthermore, neuropsychiatric symptoms such as anxiety and depression can be treated with antipsychotics, whereas cognitive decline (for example) cannot. Future iterations of our modeling framework should include these considerations as relevant data become available.

Third, the estimates for costs and utilities were derived from a burden-of-illness (BOI) study with increasingly smaller sample sizes of individuals with lower TFC scores. This was particularly true for societal costs, those of which for the Shoulson and Fahn III and IV health states were obtained from a sample of only 2 individuals. Relatedly, professional caregiving (i.e., nursing home) services were included in the direct medical cost category; however, the cost data were only available for a subgroup of participants who responded to the BOI's patient survey; therefore, our direct medical costs may be underestimated because they fail to capture the cost of professional caregiving services for a subgroup of the population. We accounted for these parameter uncertainties by modeling the full ranges of possible values, derived from each estimates' standard deviation, in sensitivity analyses.

Fourth, we did not consider safety impacts of DMTs because they are currently unknown; we assume that any DMT that eventually makes it to market will have a favorable risk-benefit profile appropriate for the indicated population; however, trial-observed major adverse events may have important impacts on cost-effectiveness estimates. Last, our results focused on a 40-year-old population only. Younger people with HD who may be years away from presenting signs and symptoms of disease may benefit from future DMTs; however, the ethical considerations^36^ and risk-benefit profile of treating these individuals is beyond the current scope of this exploratory analysis. Ultimately, our model could be adapted by future researchers to consider treatment prioritization of different age groups, CAG repeats, and other factors.

In conclusion, our novel HD-modeling framework estimates HD progression over a lifetime and the lifetime costs and QALYs. We showed that the health benefits and value of a novel DMT increase as the DMT efficacy increases; however, the tradeoffs between longer life expectancy and greater cost and prolonged time spent with a lower quality of life should be considered in future health economic evaluations of forthcoming treatments. Our framework can be used for future HD cost-effectiveness models as clinical trial evidence becomes available.

**TAKE-HOME POINTS**

→ Huntington disease (HD) is a rare, inherited, neurodegenerative disorder characterized by progressive motor symptoms including involuntary choreatic movements as well as cognitive, behavioral, and psychiatric changes.
→ Potential disease-modifying treatments (DMTs) are in development, but no clinical trial data are currently available.
→ In anticipation of future DMTs for HD, we aimed to estimate the long-term natural history of HD progression and explore the efficacy and potential value of hypothetical DMTs to treat HD using a decision-analytic modeling framework.
→ We estimated that the health benefits and value of a novel DMT increase as the DMT efficacy increases; however, the tradeoffs between longer life expectancy and greater cost and prolonged time spent with a lower quality of life should be considered in future health economic evaluations of forthcoming treatments.
→ Our framework can be used for future HD cost-effectiveness models as clinical trial evidence becomes available.

## Appendix Authors

**Table:**

Name	Location	Contribution
Gregory F. Guzauskas, MSPH, PhD	The Comparative Health Outcomes, Policy, and Economics Institute, Department of Pharmacy, University of Washington, Seattle, WA and HCD Economics, Daresbury; Huntington's Disease Centre, UCL Queen Square Institute of Neurology, London, United Kingdom	Drafting/revision of the manuscript for content, including medical writing for content; major role in the acquisition of data; study concept or design; analysis or interpretation of data
Sarah J. Tabrizi, MD, PhD	The University of Iowa Health Care, Iowa City, IA	Drafting/revision of the manuscript for content, including medical writing for content; study concept or design; analysis or interpretation of data
Jeffrey D. Long, PhD	European Huntington Association, Moerbeke Waas, Belgium	Drafting/revision of the manuscript for content, including medical writing for content; study concept or design; analysis or interpretation of data
Astri Arnesen	Clinical Department, CHDI Management/CHDI Foundation, Princeton, NJ	Drafting/revision of the manuscript for content, including medical writing for content; analysis or interpretation of data
Jamie L. Hamilton, PhD	Department of Neurology, Vanderbilt University Medical Center, Nashville, TN	Drafting/revision of the manuscript for content, including medical writing for content; analysis or interpretation of data
Daniel O. Claassen, MD	HCD Economics, Daresbury, United Kingdom	Drafting/revision of the manuscript for content, including medical writing for content; analysis or interpretation of data
Lorraine R. Munetsi, MSc	Huntington's Disease Centre, UCL Queen Square Institute of Neurology, London, United Kingdom	Major role in the acquisition of data; study concept or design; analysis or interpretation of data
Shahid Malik, MSc	Huntington's Disease Centre, UCL Queen Square Institute of Neurology, London, United Kingdom	Major role in the acquisition of data; study concept or design; analysis or interpretation of data
Idaira Rodríguez-Santana, PhD	Huntington's Disease Centre, UCL Queen Square Institute of Neurology, London, United Kingdom	Drafting/revision of the manuscript for content, including medical writing for content; analysis or interpretation of data
Talaha M. Ali, MD	uniQure Inc., Lexington, MA	Drafting/revision of the manuscript for content, including medical writing for content; study concept or design; analysis or interpretation of data
Frank Zhang, MD, MPH	uniQure Inc., Lexington, MA	Drafting/revision of the manuscript for content, including medical writing for content; study concept or design
